# Blood immunophenotyping of multiple sclerosis patients at diagnosis identifies a classical monocyte subset associated to disease evolution

**DOI:** 10.3389/fimmu.2024.1494842

**Published:** 2025-01-08

**Authors:** Stéphane Rodriguez, Laura Couloume, Juliette Ferrant, Nicolas Vince, Marion Mandon, Rachel Jean, Celine Monvoisin, Simon Leonard, Simon Le Gallou, Nayane S. B. Silva, Sonia Bourguiba-Hachemi, David Laplaud, Alexandra Garcia, Romain Casey, Helene Zephir, Anne Kerbrat, Gilles Edan, Emmanuelle Lepage, Eric Thouvenot, Aurelie Ruet, Guillaume Mathey, Pierre-Antoine Gourraud, Karin Tarte, Celine Delaloy, Patricia Amé, Mikael Roussel, Laure Michel

**Affiliations:** ^1^ Institut National de la Santé et de la Recherche Médicale (INSERM), Unité Mixte de Recherche U1236, Université Rennes, Etablissement Français du Sang Bretagne, LabEx IGO, Rennes, France; ^2^ Pole Biologie-Centre Hospitalier Universitaire (CHU) Rennes, Rennes, France; ^3^ Institut National de la Santé et de la Recherche Médicale (INSERM), Centre Hospitalier Universitaire (CHU) Nantes, Nantes University, Center for Research in Transplantation and Translational Immunology, UMR 1064, Nantes, France; ^4^ São Paulo State University, Molecular Genetics and Bioinformatics Laboratory, School of Medicine, Botucatu, Brazil; ^5^ Service de Neurologie, Centre Hospitalier Universitaire (CHU) Nantes, CRC-SEP Pays de la Loire, CIC 1413, Nantes, France; ^6^ Lyon University, University Claude Bernard Lyon 1, Lyon, France; ^7^ Hospices Civils de Lyon, Neurology Department, Sclérose en Plaques, Pathologies de la Myéline et Neuro-Inflammation, Bron, France; ^8^ Observatoire Français de la Sclérose en Plaques, Centre de Recherche en Neurosciences de Lyon, INSERM 1028 and CNRS UMR 5292, Lyon, France; ^9^ EUGENE DEVIC EDMUS Foundation against Multiple Sclerosis, State-Approved Foundation, Bron, France; ^10^ Lille University, Inserm U1172, Lille University Hospital, Lille, France; ^11^ Neurology Department, Rennes Clinical Investigation Centre, Rennes University Hospital-Rennes University-Institut National de la Santé et de la Recherche Médicale (INSERM), Rennes, France; ^12^ Department of Neurology, Nimes University Hospital, Nimes, France; ^13^ Institut de Génomique Fonctionnelle, UMR5203, Inserm 1191, Université de Montpellier, Montpellier, France; ^14^ Neurocentre Magendie, Institut National de la Santé et de la Recherche Médicale (INSERM) U1215, Bordeaux, France; ^15^ CHU de Bordeaux, Department of Neurology, Bordeaux, France; ^16^ Department of Neurology, Nancy University Hospital, Nancy, France; ^17^ Université de Lorraine, Inserm, INSPIIRE, Nancy, France

**Keywords:** multiple sclerosis, cerebrospinal fluid, classical monocyte, disability, antigen presentation

## Abstract

**Introduction:**

Myeloid cells trafficking from the periphery to the central nervous system are key players in multiple sclerosis (MS) through antigen presentation, cytokine secretion and repair processes.

**Methods:**

Combination of mass cytometry on blood cells from 60 MS patients at diagnosis and 29 healthy controls, along with single cell RNA sequencing on paired blood and cerebrospinal fluid (CSF) samples from 5 MS patients were used for myeloid cells detailing.

**Results:**

Myeloid compartment study demonstrated an enrichment of a peculiar classical monocyte population in 22% of MS patients at the time of diagnosis. Notably, this patients’ subgroup exhibited a more aggressive disease phenotype two years post-diagnosis. This monocytic population, detected in both the CSF and blood, was characterized by CD206, CD209, CCR5 and CCR2 expression, and was found to be more frequent in MS patients carrying the HLA-DRB1*15:01 allele. Furthermore, pathways analysis predicted that these cells had antigen presentation capabilities coupled with pro-inflammatory phenotype.

**Discussion:**

Altogether, these results point toward the amplification of a specific and pathogenic myeloid cell subset in MS patients with genetic susceptibilities.

## Graphical Abstract

Multiple sclerosis patients were included at diagnosis, for mass cytometry or single-cell RNA sequencing studies (scRNA-seq). Differential abundance analysis on cytof data indicated the exclusive blood enrichment in circulating myeloid cells co-expressing CD14, CCR5, CD206, and CD209 in a group of MS patients. Characterization of these patients demonstrated a significantly higher proportion of HLA-DRB1*15-01 patients allotype and a worsen outcome after 2 years follow up compared to other patients. scRNA-seq analysis confirmed the pathogenic potential of this monocyte subset through their definition as antigen presenting pro-inflammatory cells.
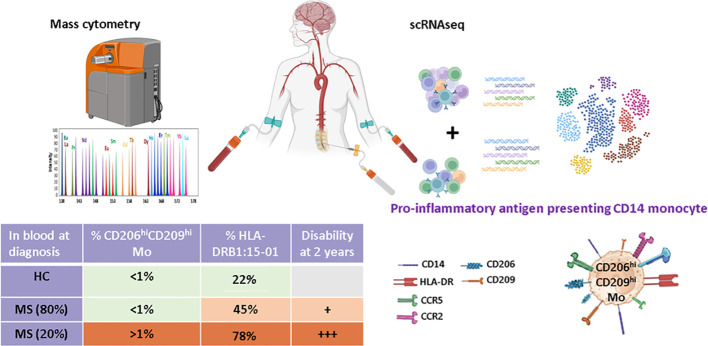

## Introduction

Relapsing-remitting multiple sclerosis (RRMS) is a demyelinating autoimmune disease characterized by chronic inflammation of the central nervous system (CNS). A complex interplay between immune cells both outside (1–3) and locally within the CNS (4, 5) dictates immune cells’ capacity to infiltrate the CNS and to polarize them into pathogenic pro-inflammatory cells. This is well illustrated with blood myeloid cells infiltrating the CNS and especially monocytes. Monocyte is a heterogeneous subset usually defined by CD14 and CD16 surface molecule expression comprising CD14^++^ CD16^−^classical monocytes (cMo), CD14^++^ CD16^++^ intermediate monocytes (intMo), and CD14^−^ CD16^++^ non-classical monocytes (ncMo). All these subsets can traffic to tissue lesions (6, 7) while microglial cells and the border-associated macrophages (BAMs) are associated with tissue-derived myeloid cells in human CSF (8). Infiltrating monocytes can acquire the dendritic cell marker CD209 following transmigration across a blood-brain barrier (BBB) model (9). Although data about human monocytes fate in MS CNS are limited, they are more abundant in the experimental autoimmune encephalomyelitis (EAE) mice model of MS. Beyond subsets mentioned earlier, tissue-infiltrating monocytes were demonstrated to alternatively differentiate in monocyte-derived dendritic cells (moDC) (10), macrophages (moMac) (11, 12), or monocytic myeloid-derived suppressor cells (m-MDSC) (13) once reaching the CNS, having either a detrimental (14) or protective role (15). There, the capacity of monocyte-derived cells to impact disease course lay on their antigen presentation propensity, their secretome profile, and phagocytic capacity (11, 16, 17), making them a valuable therapeutic target. In line, the blockade of myeloid cells trafficking from the periphery to the CNS, specifically through targeting Ninjurin-1 or more broadly through anti-VLA-4 usage, demonstrated efficacy in controlling EAE and RRMS CNS inflammation (18, 19).

Given the demonstrated role of monocytes in RRMS and their peripheral origin, many have tried to study them by standard flow cytometry within peripheral blood mononuclear cells (PBMCs). Although most studies report an increase in cMo frequency in RRMS patients, it is more controversial concerning ncMo (20–24). These discrepancies may be related to the cohorts used, the markers assessed, and/or the analysis method, pointing to the lack of robust and exhaustive characterization of the peripheral myeloid compartment in RRMS patients. Finally, although m-MDSCs abundance and monocyte/lymphocyte ratios in patients at diagnosis were correlated to higher disability overtime (25, 26) few carefully assessed the link between myeloid compartment composition at the early disease stage and individual outcome.

In this study, we took advantage of a highly characterized cohort of RRMS patients to perform mass cytometry on PBMCs sampled at diagnosis. Unsupervised analysis on the myeloid compartment allowed us to identify a specific population of classical monocytes expressing CD209 and CD206 enriched in some MS patients. This increased frequency defined a patient’s subgroup highly enriched in HLA-DRB1*15:01 individuals who displayed a poorer outcome 2 years post-diagnosis. Characterization of an equivalent monocytic population by scRNA-seq on paired CSF and blood cells from unrelated MS patients together with pathway enrichment analysis indicated that these cells are present in both CSF and blood, have a proinflammatory profile, and have a higher propensity to process and present antigens compared to other classical monocytes.

## Material and methods

### Cohorts

This study was registered and approved by the Ethics Committee of Rennes Hospital (notice n° 20.05). MS patients included in this work were extracted from the OFSEP (Observatoire Français de la Sclérose en Plaques) MS French registry (27–30), www.ofsep.org. All participants provide written informed consent for participation. In accordance with the French legislation, OFSEP was approved by both the French data protection agency (*Commission Nationale de l’ Informatique et des Libertés* [CNIL]; authorization request 914066v3) and a French ethical committee (*Comité de Protection des Personnes* [CPP]: reference 2019-A03066-51), and the present study was declared compliant to the MR-004 (*Méthodologie de reference 004*) of the CNIL.

Participating centers were Rennes, Lille, Nancy, Nimes, and Bordeaux. Inclusion criteria were: (i) age > 18 years old, (ii) MS diagnosis according to McDonald 2017 criteria at the last visit (31), (iii) sampled during their first neurological episode, and (iv) with at least one visit/year during the follow-up. Progressive MS patients were excluded. At the time of PBMC or CSF sampling, all MS patients included were drug-naïve and so had never been treated by disease-modifying therapy (DMT). Blood samples were obtained from 65 early RRMS patients and 29 age- and sex-paired healthy controls (HCs).

Clinical details of patients enrolled in mass cytometry cohort or of the scRNA-seq study are summarized in **Tables 1**, **2**, respectively.

**Table 1:** Detailed clinical parameters from healthy controls and multiple sclerosis cohorts: (HC) healthy controls, (MS) multiple sclerosis, (EDSS) expanded disability status scale, (Gd) gadolinium lesions positivity, (SC) spinal cord lesions, (CIS) clinically isolated syndrome, (CSF) cerebrospinal fluid.

Baseline variable	HC		MS						
	Total	%	Total	%	MS wo CD206^hi^ CD209^hi^ Mo (CD206^hi^ CD209^hi^ Mo < 1%)		MS w CD206^hi^ CD209^hi^ Mo (CD206^hi^ CD209^hi^ Mo ≥ 1%)		*p*-value*
					N	%	N	%	
**Total**	**29**		**60**		**47**	***78.3***	**13**	***21.7***	
**Sex**									**0.947**
Men	10	*34.5*	19	*31.6*	15	*31.9*	4	*44.4*	
Women	19	*65.5*	41	*68.4*	32	*68.1*	9	*55.6*	
**Age [median (Q1–Q3)]**									**0.829**
	30 [25.5–45.5]		31 [24–37.7]		31 [24–40]		31 [24–35.5]		
**Delay relapse onset/sampling (days)**									**0.869**
Mean ± SD	NA	NA	60.9 ± 56		58.6 ± 55		69.1 ± 60.9		
Median [Q1–Q3]	NA	NA	42.5 [14.2–93.7]		40 [15–82]		56 [5–133]		
**EDSS**									**0.869**
Mean ± SD	NA	NA	1.3 ± 1.3		1.3 ± 1.1		1.5 ± 1.4		
Median [Q1–Q3]	NA	NA	1.5 [0–2]		1 [0–2]		1.5 [0–2.5]		
**T2 lesions number ≥ 9**									**0.527**
Yes	NA	NA	35	*58.3*	26	*55.3*	9	*69.2*	
No	NA	NA	25	*41.7*	21	*44.7*	4	*30.8*	
**Gd lesions**									**0.7582**
Yes	NA	NA	31	*51.7*	25	*53.2*	6	*46.1*	
No	NA	NA	29	*48.3*	22	*46.8*	7	*53.85*	
**SC lesions**									**0.758**
Yes	NA	NA	39	*65*	30	*63.8*	9	*69.2*	
No	NA	NA	21	*35*	17	*36.2*	4	*30.8*	
**CIS type**									**0.737**
Motor-Brainstem	NA	NA	19	*31.6*	14	*29.8*	5	*38.5*	
Sensitive-optical nerve	NA	NA	41	*68.4*	33	*70.2*	8	*62.5*	
**CSF oligoclonal bands (15NA)**			**45**		**33**		**12**		**0.741**
Yes	NA	NA	40	*88.9*	29	*87.9*	11	*91.6*	
No	NA	NA	5	*11.1*	4	*12.1*	1	*8.4*	

When indicated, the mean and standard deviations (SD) are displayed as well as the median with Q1-Q3 interquartile in brackets. *p-values: correspond to Mann-Whitney statistical test *p*-value. NA, not applicable. Percentage are in italic and values in bold.

**Table 2:** Detailed clinical parameters from scRNAseq cohort: *(HC)* healthy controls, *(MS)* multiple sclerosis, *(EDSS)* expanded disability status scale, *(Gd)* gadolinium lesions positivity, *(SC)* spinal cord lesions, *(CIS)* clinically isolated syndrome, *(CSF)* cerebrospinal fluid.

Baseline variable	RRMS	
	Total	%
**Total**	**5**	
Sex		
Men	1	*20*
Women	4	*80*
Age [median (Q1-Q3)]		
	35 [24–43]	
Delay relapse onset/sampling (days)		
Mean ± SD	56.8 ± 32.76	
Median [Q1–Q3]	45 [28.5–91]	
EDSS		
Mean ± SD	1.5 ± 0.58	
Median [Q1–Q3]	1.5 [1–2]	
T2 lesions number ≥ 9		
Yes	2	*40*
No	3	*60*
Gd lesions		
Yes	4	*80*
No	1	*20*
SC lesions (1NA)		
Yes	2	*50*
No	2	*50*
CIS type		
Motor-Brainstem	2	*40*
Sensitive-Optical nerve	3	*60*
CSF oligoclonal bands		
Yes	5	*100*
No	0	*0*

When indicated, the mean and standard deviations (SDs) are displayed as well as the median with Q1–Q3 interquartile in brackets. Percentage are in italic and values in bold.

### Blood samples processing

Blood was collected in heparin tubes for a total of 30 to 50 mL. The same volume of phosphate buffered saline (PBS) was added to the blood and diluted blood was then gently deposited on 20 mL Lymphoprep (Eurobio scientific, Ref: CMSMSL01-0U), followed by 20 min centrifugation at 1000 g with no brakes. Lymphocytes ring was then collected, washed, plaquettes were removed (two centrifugations 10 min, 200 g, 4°C with no brakes), and red blood cells were lysed by the use of Easylyse (Dako) (10 min at room temperature). Peripheral blood mononuclear cells (PBMCs) were then counted and viability assessed by trypan blue staining. An average of 40 million cells was obtained per donor and banked in Foetal Calf Serum (FCS) 10% dimethylsulfoxide in two cryovials containing 20 million cells each and stored in liquid nitrogen for subsequent usage. When PBMC samples were thawed for experimentation purposes, cell viability and cell count were obtained by the use of a Nucelocounter NC-200 (Chemotec, 3450 Allerod, Denmark) device. Cell viability ranged from 85% to 96%.

### Plasma collection

Heparin blood tubes were pooled, and 20 mL of blood was used to get plasma. Blood was centrifugated (680 g, 5 min) and plasma collected for banking at −80°C.

### CSF samples processing

Five milliliter of CSF was obtained by lumbar puncture; samples were immediately processed by centrifugation (450 g, 5 min), and the supernatant was stored at −80°C while cells were counted and cell viability assessed by trypan blue staining. Cell viability ranged from 90% to 98%. On average, 25,000 cells were obtained per donor. Cells were then immediately used for scRNA-seq experiments.

### Mass cytometry

Frozen PBMCs from MS patients and sex- and age-matched HC were processed for cytofin staining as previously described (32, 33). A minimum of 2 million cells were used for staining. Antibodies used for cell staining and listed in **Supplementary Table S1** were purchased in either metal-labeled (Fludigm antibodies) or uncoupled format. Antibody conjugation to a metal with the Maxpar Antibody Labeling Kit (Fluidigm) and subsequent titration were done prior to the staining procedure and according to manufacturer protocol. Briefly, cells from frozen PBMCs were counted, and Cisplatin cell ID staining was done to assess cell viability. In the next step, membrane markers of interest were labeled with a cocktail of dedicated antibodies, while subsequent cell fixation and permeabilization with Fix Perm Buffer (Miltenyi Ref: 130093142) allowed the assessment of intracellular marker expression. Intra-cellular staining was therefore done, followed by iridium labeling to discriminate singlets from doublets during analysis. Finally, suspensions of fixed cells were banked at −80°C until acquisition on the Helios™ System (Fluidigm) from the CyPS plateform (Paris Pitié Salpêtrière).

### Cytof data analysis

FCS files obtained from the platform were first processed for bead-based normalization on EQ-Beads (Fluidigm) through the use of the R package premessa (https://github.com/ParkerICI/premessa). Such normalized FCS then served as input for sample cleaning from debris and doublets (DNA1 vs. DNA2), from dead cells (DNA1 vs. Cisplatin), and beads (Ce140D1 vs. DNA1) within the Cytobank cloud-based platform (Cytobank, Inc). Dimensional reduction was performed on each file separately according to the viSNE algorithm and settings defined previously (34) (perplexity = 30; iterations = 5000; theta = 0.45). Clusters corresponding to myeloid cells were delineated based on lineage marker expression (CD45^+^CD3^-^CD19^-^CD36^+^HLA-DR^+^) and then exported for deep analysis with the help of the Catalyst package on R (https://rdrr.io/bioc/CATALYST). In total, more than 11 million myeloid cells were analyzed, ranging from 278 to 312,260 cells retrieved per patient. The cell clustering process was done with all markers except those used for lineage discrimination through the FlowSOM algorithm and with the following parameters: self-organizing map = 20 × 20 and maxK = 30 (maximum number of meta clusters to evaluate). Each FlowSOM-defined cluster was evaluated and either kept untouched for further analysis, merged with phenotypically similar clusters, or removed when related to other cell lineage residual contamination. Retained clusters were highlighted on UMAP dimensional reduction based on the same markers as those used for FlowSOM clustering. Differential frequencies of major monocyte subsets were assessed through the Mann and Whitney test. Clusters’ differential abundances between individual groups were analyzed through a generalized linear mixed model (GLMM) and Benjamini-Hochberg adjustment, while differential marker expression between HC, CD206^hi^, and CD209^hi^ cMo-enriched patients and not-enriched patients was tested with multiple ANOVA and a Tukey *post hoc* test. Results were considered significant when adj *p* < 0.05.

### scRNA-seq sequencing

Paired CSF and blood collected from MS patients at their first neurological episode were used for scRNA-seq experiments. Although CSF cells were processed freshly, PBMCs get a freezing/thawing cycle before use. A mean of 25,000 cells from CSF (corresponding to all cells) and the equivalent amount of paired PBMCs were loaded in the Chromium 10× (10× Genomics, Pleasanton, CA, USA) for single-cell capture and barcoding. Libraries were prepared according to the manufacturer’s protocol with the 10× 5’ kit (Chromium Next Gem Single Cell 5’ reagent kit v1.1). Libraries were processed using NovaSeq 6000 (Illumina, San Diego, CA), with a depth of 50,000 reads/cell and a paired sequencing of 28 nucleotides in R1 and 91 in R2. Sequenced were then aligned with Cellranger v6.1.1 in intron inclusion mode on the Human GRCh38 scRNA-seq optimized transcriptomic reference v1.0 as in Pool et al. (35) The median number of genes retrieved per cell ranged from 1.651 to 1.981 in CSF while it varied from 812 to 3,014 in peripheral blood. Quality controls were done on each sample individually, and cells displaying either several genes lower than 400 or higher than 4,000, several UMI over 15,000, a mitochondrial cell read ratio higher than 10%, or a ribosomal gene’s frequency lower than 8% were filtered out, as clusters predicted to comprise mainly doublets through singleCellTK package v2.8.0.

### scRNA-seq data processing

Filtered datasets from each sample were merged and log normalized before data integration with the FindIntegrationAnchors and IntegrateData functions from Seurat v4.3.0 on the first 20 correlation components. Immunoglobulin and T-cell receptor genes were removed for the integration step only. PCA analysis was then done on an integrated dataset and used for UMAP processing and cell clustering (20 nearest neighbors, resolution 0.4). Cell subset labeling resulted from the concordance between several analyses. Briefly, differentially expressed genes between clusters were obtained through the FindAllMarkers function from the Seurat package with test.use set to “wilcox.” These lists were then used in EnrichR to predict cell type. In addition, gene signature characteristics from cell lineage and obtained from literature (36) were used to assess the enrichment score of these signatures in all clusters previously defined via the ModuleScore function from Seurat. Finally, SingleR v2.0.0 was used to automatically assign labels to either cells or clusters based on signatures managed by celldex package v1.8.0. Reference index tested were HumanPrimaryCellAtlasData, BlueprintEncodeData, DatabaseImmuneCellExpressionData, MonacoImmuneData and NovershternHematopoieticData. The same strategy was used to label clusters at any step.

Once cell lineage subsets were defined, myeloid cells were sorted, and the newly generated dataset was integrated according to the Harmony algorithm from the Harmony package v0.1.1. Newly defined clusters (15 nearest neighbors, resolution = 0.15), were then labeled as previously detailed, and this whole process was repeated to retrieve classical monocyte populations (15 nearest neighbors, resolution = 0.5). CD206^hi^ CD209^hi^ cMo signature scoring resulted from Module Score function processing. To decipher specific pathways characterizing the different clusters comprised among classical monocytes, pathways from MSigDB Hallmark and Kegg were used to assess specific pathway enrichment at the single cell level with the use of the AUCell and GSEABase packages v1.20.2 and 1.60.0, respectively. Differential enrichments in gene signatures were appreciated following a comparison of CSF and blood compartments. Finally, an interactome study was done on CSF cell subsets through the use of CellChat package v1.6.1.

### Genotyping, imputation, and scoring

DNA was extracted from PBMCs obtained from 50 patients from the mass cytometry cohort. The samples underwent genotyping using the Affymetrix PMRA chip array. Standard quality controls on individuals and SNPs were performed using Plink (37). From 852,860 SNPs, quality control resulted in a remainder of 414,387 SNPs. SNPs were excluded based on the following criteria: non-autosomal (*N* = 34,049), deviation from Hardy-Weinberg equilibrium (*N* = 1,800), low genotyping (*N* = 11,848), and low frequency [minor allele frequency (MAF) < 0.01, *N* = 344,210]. Some SNPs were below both genotyping and frequency thresholds. We then performed SNP imputation. First, we converted plink files into vcf files using bcftools (38), next, we used Topmed to impute our dataset using default parameters (imputation.biodatacatalyst.nhlbi.nih.gov) (39). We obtained 9,073,739 high-confidence (>0.8) SNPs after imputation.

From these imputed SNPs, we calculated the MSGB (MS Genetic Burden) (40). This polygenic MS risk score follows a log-additive model: . MSGB was calculated with 195 SNPs extracted from the latest published GWAS on MS (41).

In addition, we performed *HLA* imputation using the HIBAG R package (42) and our in-house reference panel built with the 1000 Genomes projects data (43, 44). This allowed us to determine which patient carried the HLA-DRB1*15:01 allele.

### Global age-related multiple sclerosis severity

gARMMS was calculated through EDSS scores ranking based on the patient’s age at the time of assessment (45), in this study: 24 months following diagnosis. The frequency of patients with a gARMSS score higher than 5 was calculated among the patient’s groups. Mann-Whitney test was done to assess significant differences between patients’ groups.

### Neurofilaments, cytokines, and chemokines

Neurofilament light chain (R-PLEX F217X-3), sIL2RA, IL-15, CXCL10, CCL2, CCL20, and CXCL12 (U-PLEX biomarkers group 1 custom) content in plasma samples was assessed with a QuickPlex SQ 120MM Reader (Society Meso Scale Discovery, Rockville, MD). Undiluted samples were deposited on dedicated coated plates, and the standard procedure was followed according to the manufacturers’ protocol. Mann-Whitney test was done to assess significant differences between patients’ groups.

### Statistical analysis

Unless specified, statistical analyses were done using GraphPad Prism software, version 8.4. A two-tailed Mann-Whitney test was performed to compare two independent groups or more than two independent groups. *P*-values ≤ 0.05 were considered significant. A Fisher exact test was used to test proportional differences; *p*-values ≤ 0.05 were considered significant.

### Data accessibility

Data are deposited on EGA on accession number: EGA50000000296.

## Results

### Blood of RRMS patients is enriched in a specific subset of myeloid cells

**Figure 1:**
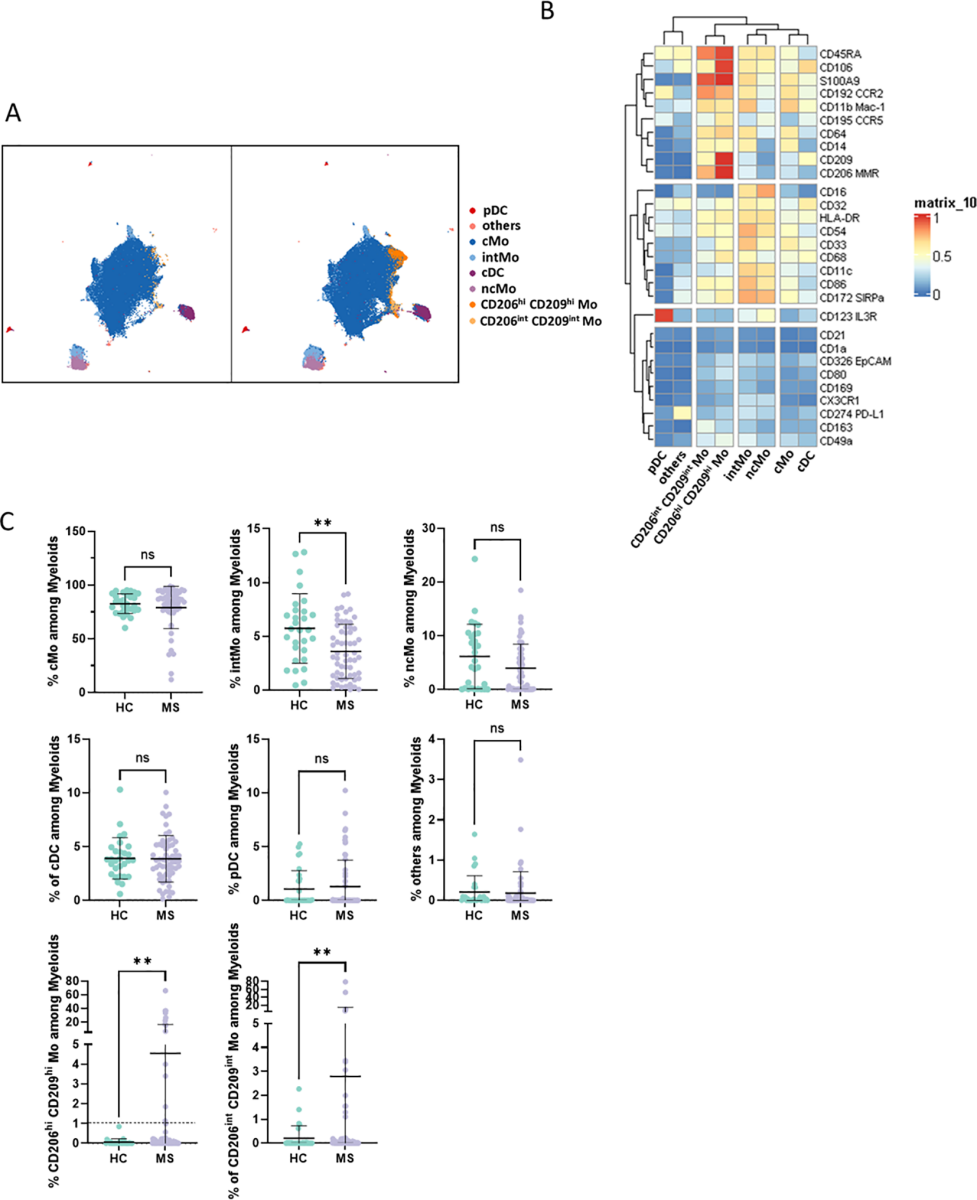
**(A)** UMAPs illustrating the myeloid clusters retrieved following FlowSOM unsupervised analysis in HC (left) and MS donors (right). **(B)** Heatmap summarizing markers expression scaled by row among clusters defined through unsupervised analysis. **(C)** Dotplots illustrating myeloid subsets frequencies among myeloid cells. Mann-Whitney test was used to determine statistical differences with ns: not significant, **p < 0.01.

To explore patients’ myeloid phenotypes, PBMCs from 60 highly characterized RRMS patients (MS) sampled at diagnosis together with 29 samples from age- and sex-matched HCs were processed for mass cytometry and unsupervised analysis (**Table 1**). viSNE visualization of high-dimensional single-cell data based on the t-Distributed Stochastic Neighbor Embedding (t-SNE) algorithm of each sample eased the delineation of myeloid cells among circulating cells based on lineage markers CD19, CD16, CD36, and CD3 expression (**Supplementary Figure S1A**). From these, no differences in frequencies of CD3 T, B lymphocytes, or myeloid cells were observed between MS and HC donors (**Supplementary Figure S1B**). To further detail myeloid population composition, a myeloid subset from each sample was isolated and processed with FlowSOM algorithm to cluster these cells, and then specific identities were assigned to each cluster based on their associated cell phenotype. Eight clusters were considered using this strategy and plotted on Uniform Manifold Approximation and Projection (UMAP) (**Figure 1A**). Among these clusters and according to their respective CD14/CD16 expression (**Figure 1B**), cMo, intMo, and ncMo were identified. Interestingly, in addition to these prototypical phenotypes, we highlighted two subsets of monocytes: CD206^hi^ CD209^hi^ Mo and CD206^int^ CD209^int^ Mo that expressed CD14 but not CD16 and clustered separately from cMo due to their strong expression of specific markers (**Figure 1B**). In addition, conventional dendritic cells (cDC) were defined through the expression of CD11c in the absence of CD14 and CD16 expression, plasmacytoid dendritic cells (pDC) were distinguished by high CD123 expression, while remaining cells (others) were characterized through the poor expression of most of the markers assessed but intermediate levels of CD11c, CD11b, and high levels of PD-L1. When frequencies of the different monocytic clusters were compared between HC and MS patients, significant differences were observed with a decreased intMo frequency in MS patients (mean HC vs. MS: 5.74% ± 3.23 vs. 3.6% ± 2.52, *p* = 0.027), while CD206^hi^ CD209^hi^ Mo (mean HC vs. MS: 0.06% ± 0.16 vs. 4.52% ± 12.1, *p* = 0.01) and CD206^int^ CD209^int^ Mo (mean HC vs. MS: 0.21% ± 0.51 vs. 2.79% ± 12.1, *p* = 0.0014) were increased (**Figure 1C**).

### Enriched myeloid cells display characteristics of activated and tissue-trafficking classical monocyte

**Figure 2:**
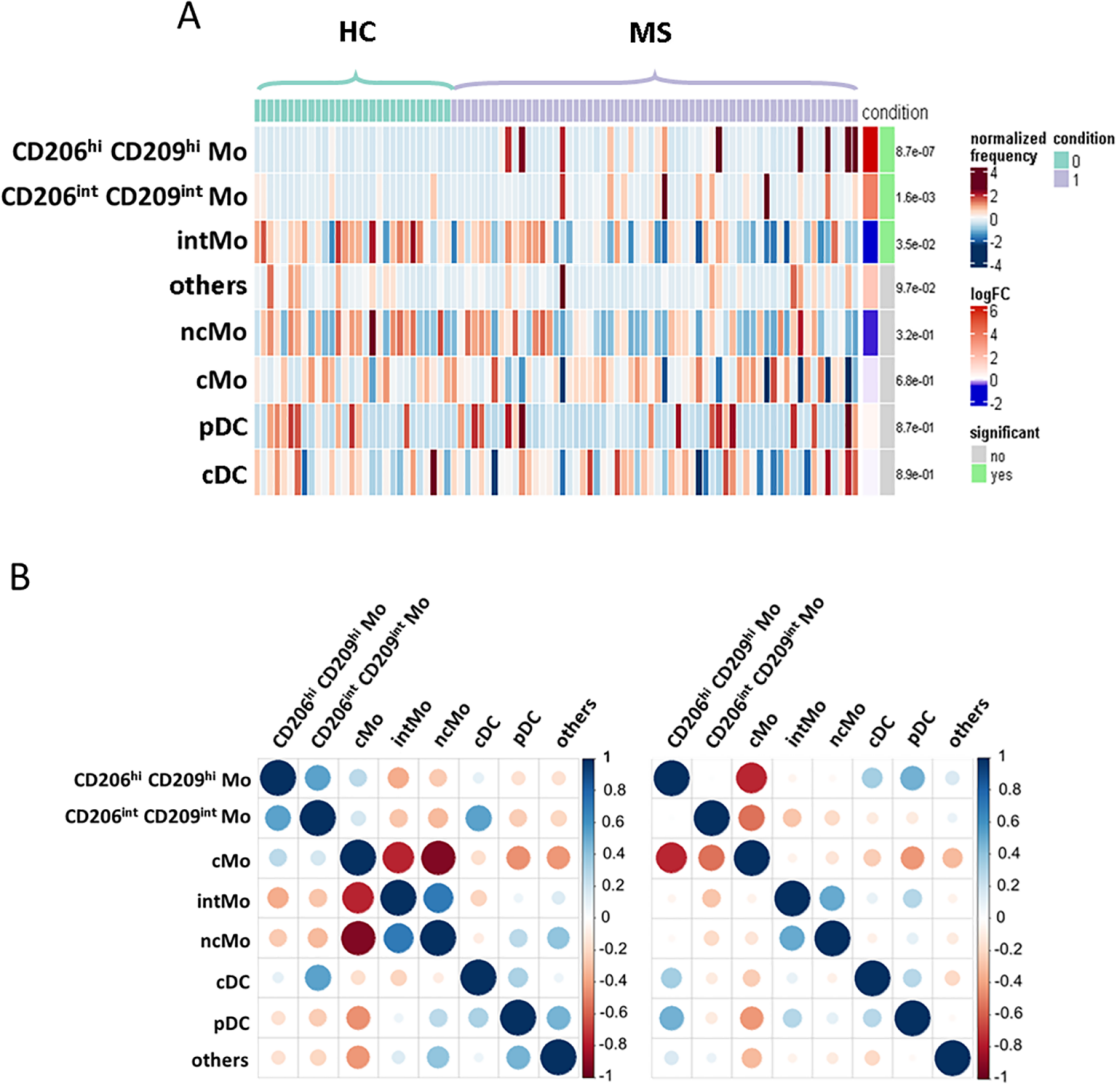
Enriched myeloid cells display characteristics of activated and tissue resident classical monocyte. **(A)** Heatmap figuring cluster differential abundance between HC and MS donors, with each bar within a cluster representing a donor and color is depending on differential enrichment. Differential abundance significance was tested through generalized linear mixed model and *p*-values for each cluster are indicated on the right. **(B)** Correlation matrix depicting correlation between myeloid subsets frequencies among HC donors (left) and MS patients (right).

To confirm CD206^hi^ CD209^hi^ Mo enrichment in MS patients, differential myeloid cluster abundance was tested by a generalized linear mixed model. In line with frequencies analysis, CD206^hi^ CD209^hi^ Mo abundancy was significantly associated with MS status (*p* = 8.7e-07) as CD206^int^ CD209^int^ Mo to a lesser extent (*p* = 1.6e-03), while intMo were more abundant in HC (*p* = 0.035) (**Figure 2A**). A closer look at CD206^hi^ CD209^hi^ Mo frequency indicated that such enrichment was occurring in only a part of MS patients while being virtually absent from HC (**Figure 2A**). Accordingly, a 1% threshold of CD206^hi^ CD209^hi^ Mo frequency among myeloid cells allowed to discriminate HC from MS patients, and among MS patients, those with CD206^hi^ CD209^hi^ Mo enrichment: frequency ≥ 1% (MS wo CD206^hi^ CD209^hi^ Mo) from those with no enrichment: frequency < 1% (MS wo CD206^hi^ CD209^hi^ Mo) (**Figure 1C**, lower left panel: dashed line illustrates the 1% threshold and **Supplementary Figure S2A**: illustrative myeloid composition according to the donors’ status). To get insights about CD206^hi^ CD209^hi^ Mo enrichment in MS patients regarding the other myeloid subsets, we performed a correlation analysis on myeloid population frequencies in HC and MS patients. Correlation matrix patterns between HC and MS donors were found to be highly different, with a strong and significant anti-correlation between CD206^hi^ CD209^hi^ Mo and cMo frequencies in MS patients that was absent from HC (**Figure 2B**), suggesting that cMo and CD206^hi^ CD209^hi^ Mo subsets were intimately related. Further, the high HLA-DR, CD86, and CD45RA expression by CD206^hi^ CD209^hi^ Mo together with high levels of CCR5, CCR2, and CD106 markers compared to cMo cells (**Figure 1B**) pointed out an active pro-inflammatory profile (46, 47) associated with trafficking abilities toward inflamed tissues (3, 7). In addition to these markers, CD206^hi^ CD209^hi^ Mo cells intriguingly expressed CD206 (MMR/MRC1) and CD209 (DC-SIGN), while these molecules are more classically found on monocyte-derived tissue resident cells (9, 48). To ascertain the co-expression of these markers, we assessed the percentage of cells expressing these discriminating molecules within their related cluster and confirmed that CD206^hi^ and CD209^hi^ Mo cells were a pure population co-expressing CD206 and CD209 together with the mentioned markers (**Supplementary Figure S2B**). Altogether, these results demonstrated that CD206^hi^ CD209^hi^ Mo are monocytes that differed from the classical monocyte archetypical phenotype via the upregulation of inflammatory and trafficking markers. Therefore, whether CD206^hi^ CD209^hi^ Mo amplification reflects a shift of monocytic cell phenotype or a disease-linked expansion of this subset remained to be determined since no significant increase in cMo frequency was observed (mean = 82.92% ± 9.31 vs. 86.55% ± 8.17, *p* = 0.0673) (**Supplementary Figure S3A**). To know whether this phenotypic change was associated with the modulation of plasmatic cytokines/chemokines concentrations, we seek for differences in sIL2RA, IL-15, CXCL10, CCL2, CCL20, and CXCL12 plasmatic content between MS patients with CD206^hi^ CD209^hi^ Mo and MS patients without CD206^hi^ CD209^hi^ Mo. No significant differences were found between MS groups (**Supplementary Figure S3B**).

### MS patients with CD206^hi^ CD209^hi^ Mo cells present a poorer prognosis

**Figure 3:**
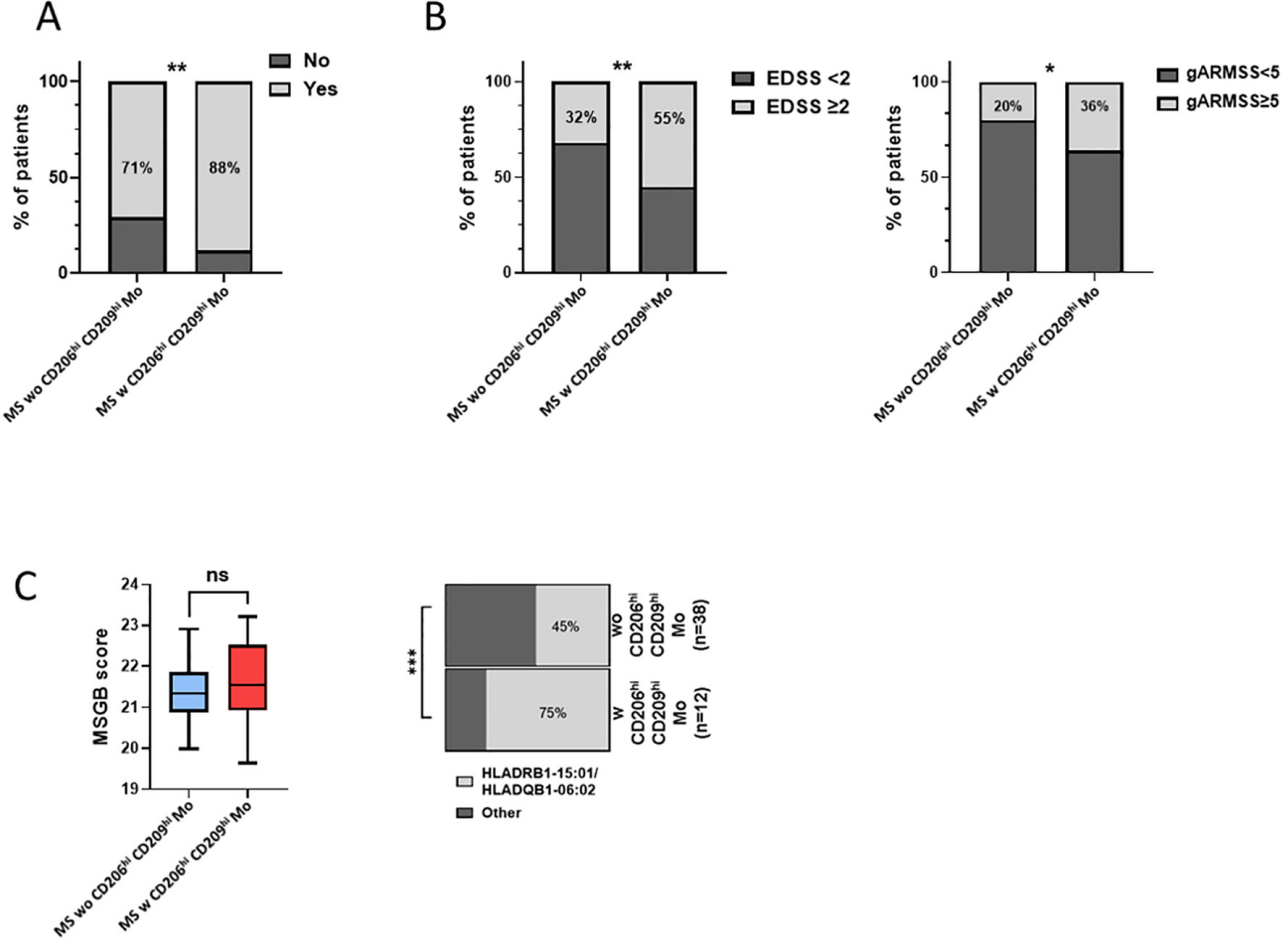
MS patients w CD206^hi^ CD209^hi^ Mo have a peculiar profile. **(A)** Histogram plot illustrating radio-clinic activity in patients displaying low (MS wo CD206^hi^ CD209^hi^ Mo) or high (MS w CD206^hi^ CD209^hi^ Mo) CD206^hi^ CD209^hi^ Mo cells frequency. Significance is based on Mann and Whitney test with **p < 0.01. **(B)** percentage of patients with EDSS ≥2 at 2 years (left). Significance is based on Mann and Whitney test with *p <0.05 and **p < 0.01. Histogram plot depicting ARMSS score ≥5 frequency among MS wo CD206^hi^ CD209^hi^ Mo and MS w CD206^hi^ CD209^hi^ Mo, 2 years following diagnosis (right). Significance is based on Fisher’s exact test with *p* = 0.0178. **(C)** Boxplot indicating MSGB patients’ score. Mann and Whitney test demonstrated no significant differences between groups (ns) (left). Histogram plot illustrating the frequency of patients HLA-DRB1*15:01 and HLA-DQB1*06:02 genes (right). Fisher’s exact test demonstrated highly significant difference with ***p < 0.001.

Next, considering that only 22% of our MS cohort displayed an enrichment in CD206^hi^ CD209^hi^ Mo cell frequency, we decided to study patients’ profiles according to CD206^hi^ CD209^hi^ Mo cell enrichment. At baseline, no significant differences on clinical and demographical variables were observed between MS patients with CD206^hi^ CD209^hi^ Mo and MS patients with CD206^hi^ CD209^hi^ Mo (**Table 1**). However, after 2 years of follow-up, a significantly higher percentage of MS patients with CD206^hi^ CD209^hi^ Mo experienced inflammatory activity (relapses and/or new T2 lesions on magnetic resonance imaging) compared to MS patients wo CD206^hi^ CD209^hi^ Mo (71% vs. 88%; *p* < 0.01) (**Figure 3A**), and significantly more MS patients with CD206^hi^ CD209^hi^ Mo presented an EDSS score ≥2 compared to MS patients wo CD206^hi^ CD209^hi^ Mo (32% vs. 55%; *p* < 0.01) (**Figure 3B**, left). Corroborating these results, we found a significantly higher proportion of patients displaying an age-related multiple sclerosis severity (ARMSS) (45) score higher than five among MS patients with CD206^hi^ CD209^hi^ Mo two years following diagnosis (*p* = 0.018) (**Figure 3B**, right), while no correlation between CD206^hi^ CD209^hi^ Mo frequency and donor age was observed (**Supplementary Figure S3C**). Further, although no differences were noticed between groups in plasmatic neurofilament light chain (Nfl) concentration (**Supplementary Figure S4**), nor in patients’ burden in genes at risk (MSGB), using the most recent associated SNPs set from the International Multiple Sclerosis Genetics Consortium (41) (**Figure 3C**, left), we found that 75% of MS patients with CD206^hi^ CD209^hi^ Mo carry either HLA-DRB1*15:01 and/or HLA-DQB1*06:02 compared to 45% in MS patients with CD206^hi^ CD209^hi^ Mo (*p* < 0.001) (**Figure 3C**, right). Altogether, these results point out a potential role of these cells in disease severity and a potential role of HLA-DRB1*15:01 haplotype in CD206^hi^ CD209^hi^ Mo higher frequency.

### CD206^hi^ CD209^hi^ Mo-like cells infiltrate RRMS patients’ CSF at diagnosis

**Figure 4:**
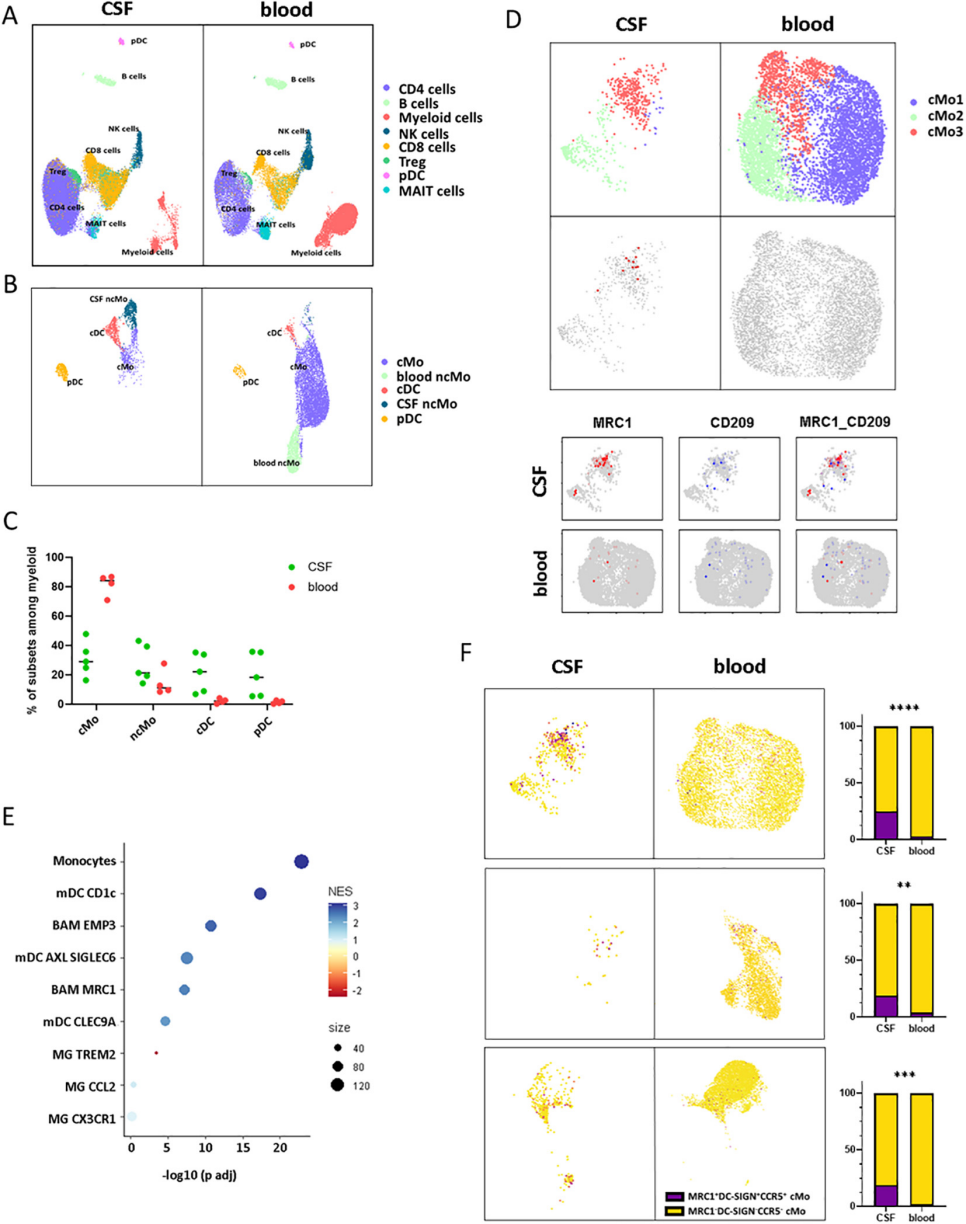
CD206^hi^ CD209^hi^ Mo-like cells can be found in RRMS CSF at diagnosis. **(A)** UMAPs representing immune cells retrieved and analyzed from scRNA-seq cohort in blood and CSF of MS patients following integration and unidentified cells removal. Clusters are labeled with cell identities according to genes expression. **(B)** UMAPS illustrating myeloid cell compartment in both CSF and blood with cluster labeling according to genes signatures. **(C)** Circle diagram displaying cell subset proportions among myeloid cells in CSF and blood with bars as median. **(D)** UMAPs depicting classical monocytes major clusters (upper panels). UMAPs illustrating *MRC1/DC-SIGN* co-expressing events in CSF and blood (middle panels). UMAPs figuring classical monocytes events expression of *MRC1*/CD206 (red) and *DC-SIGN*/CD209 (blue) transcripts in CSF and blood (lower panels). **(E)** Circle diagram displaying CD206^hi^ CD209^hi^ Mo-like cell proximity to the related myeloid subset gene signatures through GSEA. Circle color depicts normalized enrichment score and circle size: CD206^hi^ CD209^hi^ Mo-like cells/associated subset signatures overlap. **(F)** (left panels) UMAPs figuring CD206^hi^ CD209^hi^ Mo signature scoring (*CCR5, MRC1, DC-SIGN*) among classical monocyte events from our dataset (up), Esaulova dataset (middle), Ramesh dataset (lower). (Right panels) UMAP associated histogram plots depicting CD206^hi^ CD209^hi^ Mo signature positive cells frequency among classical monocytes in CSF and PB. Significance is based on Mann and Whitney test with **p < 0.01, ***p < 0.001, ****p < 0.001.

In an attempt to get further insights into CD206^hi^ CD209^hi^ Mo cell specificities and potential role in MS pathogenicity, we explored data from paired CSF and blood scRNA-seq from RRMS patients sampled at diagnosis (*n* = 5) (**Table 2**). More than 55,000 cells were recovered and analyzed for gene expression. Cell clustering based on differential gene expression allowed to discriminate major cell lineages and annotate them based on characteristic gene expression, supported by SingleR-assisted labeling while cells with an undefined identity were not represented (**Figure 4A**). This strategy allowed us to isolate myeloid cells from other circulating cells and to retrieve 2232 and 7840 myeloid cells from CSF and blood, respectively (**Figure 4B**). In line with literature (49–51), the myelocytic composition of CSF differed from the blood one, with a differential enrichment when comparing median frequencies in both cDC (22.24% of myeloid CSF cells retrieved vs. 2.18% of blood myeloid cells), pDC (18.42% vs. 1.44%), as well as in ncMo expressing CD16 (21.41% in CSF vs. 11.21% in the blood) in CSF concomitant to a decreased frequency of CD14 expressing cMo cells (29.02% in CSF vs. 84.2% in the blood) (**Figure 4C**). Considering that CD206^hi^ CD209^hi^ Mo cells express CD14 but not CD16, we first isolated cMo from other myeloid cells and performed a subset clustering. Two (cMo2 and cMo3) out of the three clusters recapitulating the cMo population were found in both CSF and blood, while the cMo1 subset was observed in the blood compartment only (**Figure 4D**, upper panels). To identify CD206^hi^ CD209^hi^ Mo among clusters, we then looked for events co-expressing the two discriminating markers: CD206 and CD209 (*MRC1* and *DC-SIGN*, respectively) at the transcript level. Although few events were strictly co-expressing *MRC1* and *DC-SIGN* (**Figure 4D** middle and lower panels), cMo clustering indicated that they were found almost exclusively in one CSF cluster: cMo3. Importantly, such events were observed in four out of five CSF samples (**Supplementary Figure S5**), avoiding individual bias. To exclude microglial contamination and confirm the monocytic identity of detected CD206^hi^ CD209^hi^ Mo-like cells, we tested through gene set enrichment analysis their transcriptomic proximity with human CNS myeloid cell subsets as defined in literature (8). CD206^hi^ CD209^hi^ Mo-like cells were found enriched in genes belonging to monocytic lineage (adjusted *p*-value = 9,78E-23) rather than BAMs (EMP3, adjusted *p*-value = 1.39E-11) or microglial cells (MG TREM2: negative enrichment score or MG CCL2, MG CX3CR1: adjusted *p*-value > 0.05) (**Figure 4E**). They were also discriminated from CD1c mDC considering *CD14* expression by CD206^hi^ CD209^hi^ Mo-like cells. Therefore, CD206^hi^ CD209^hi^ Mo-like cells are predicted as originating from circulating rather than resident myeloid cells from the CNS, in line with their presence in the peripheral bloodstream. To better quantify CD206^hi^ CD209^hi^ Mo-like cell frequency in the different compartments and to refine their identification at the RNA level despite transcript drop-out, we selected, among the best-expressed transcripts, *CCR5* as an additional CD206^hi^ CD209^hi^ Mo marker to the CD206^hi^ CD209^hi^ Mo-like gene signature (*MRC1, DC-SIGN, CCR5*). Signature scores for each event plotted on the UMAPs were retrieved, and the number of cells with positive scores (**Figure 4F** UMAPs dark dots) was assessed. A significant enrichment in our dataset of CD206^hi^ CD209^hi^ Mo-like cells was highlighted in CSF while few events were found in peripheral blood (**Figure 4F** higher panels), indicating that CD206^hi^ CD209^hi^ Mo-like cells can be observed in both CSF and blood. The presence of CD206^hi^ CD209^hi^ Mo in both blood and CSF compartments was additionally assessed in two publicly available datasets (50, 51) following the same strategy. In line with our findings, although cMo frequency was strongly decreased in CSF compared to blood in these other datasets (**Figure 4F** middle panels: Esaulova et al. dataset, lower panels: Ramesh et al. dataset), CD206^hi^ CD209^hi^ Mo-like can be identified in both compartments. Nevertheless, CD206^hi^ CD209^hi^ Mo-like cell frequency was found higher in CSF than in blood, similarly in all the datasets tested (**Figure 4F** right panels). Altogether, these demonstrated that cells with close similarities to CD206^hi^ CD209^hi^ Mo cells as *CD14, MRC1, DC-SIGN*, and *CCR5* transcript expression can be found in both peripheral blood and CSF from RRMS patients by scRNA-seq data. These are enriched in a specific monocyte cluster, and their higher frequency in CSF may suggest either (i) their higher migration capacities, (ii) their better retention/survival in CSF, (iii) and/or an increased monocyte polarization in CSF.

### CD206^hi^ CD209^hi^ Mo-like cells’ phenotypic characterization at the transcriptomic level

**Figure 5:**
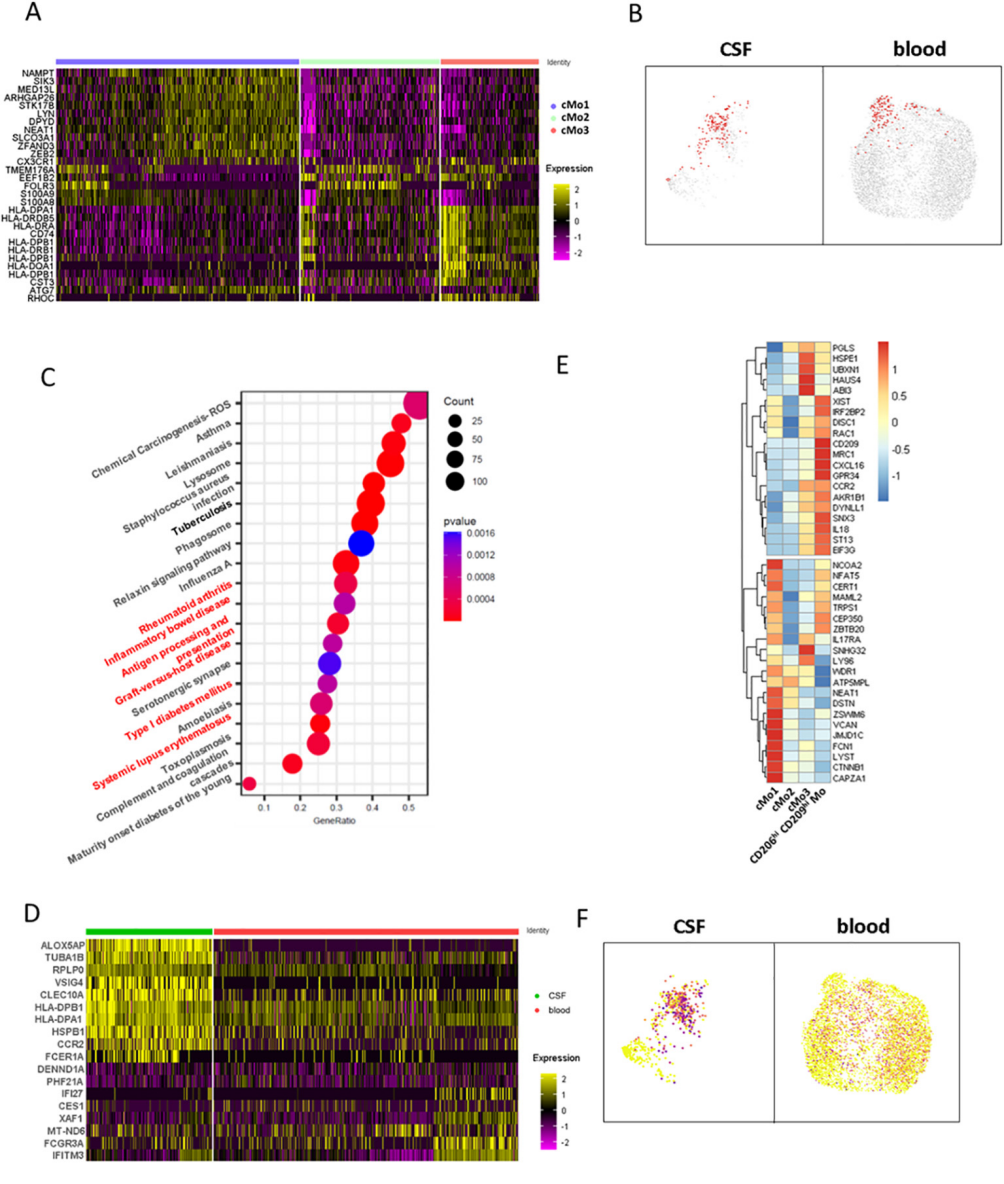
CD206^hi^ CD209^hi^ classical monocyte-like cells phenotypic characterization at the gene level. **(A)** Heatmap depicting differentially expressed genes characterizing classical monocytes major clusters defined in **Figure 4D** upper panels. **(B)** UMAPs illustrating cells enriched in the genes signature belonging to the antigen processing and presentation pathway from the Kegg database. Enriched cells are figured as red dots in CSF (left) and blood (right). **(C)** Circle diagram illustrating CSF cMo3 cluster comparison to its peripheral blood counterpart through GSEA analysis. Enriched pathways are depicted, with signature overlap with cMo3 CSF versus blood differentially upregulated genes illustrated through circle size and *p*-value as color gradient. Pathways related to immune cells activation are labeled in red. **(D)** Heatmap displaying differentially expressed genes between CSF cMo3 cluster and its peripheral blood counterpart. **(E)** Heatmap displaying top regulated genes among CD206^hi^ CD209^hi^ Mo-like cells with CD206^hi^ CD209^hi^ Mo-like cells defined by MRC1/CD209 expression in comparison to previously defined classical monocyte clusters cMo1, cMo2, and cMo3. **(F)** UMAPs illustrating CCR2 expression among classical monocytic cells.

To decipher CD206^hi^ CD209^hi^ Mo-like cells’ properties, we first considered this population defined at the cluster level according to **Figure 4D** and sought for their specifically active pathways in comparison to the other classical monocyte clusters. Cluster analysis indicated that cMo3, the cluster comprising CD206^hi^ CD209^hi^ Mo-like events, was characterized by cells displaying high *HLA* molecule expression together with higher *CD74* (**Figure 5A**). Querying events displaying antigen processing and presentation properties according to the Kegg database confirmed that the cMo3 cluster had a greater propensity to process and present antigens (**Figure 5B**, Kegg database signature-enriched events plotted in red). This capacity was also found to be significantly higher (*p* = 0.0004) when we performed prospective gene set enrichment analysis comparing the CSF cMo3 cluster to its peripheral blood counterpart (**Figure 5C**), supported by significantly higher *HLA* expression in the CSF cluster compared to the blood one (**Figure 5D**). In addition, pathways related to immune cell activation (**Figure 5C**, red labels) were found overactivated in CSF migrating cells compared to their blood counterpart, reflecting their proinflammatory phenotype. These data therefore suggest that the cMo3 cluster corresponds to a monocyte subset with antigen presentation with enhanced proinflammatory properties once in the CSF. To strengthen our findings and regarding that cMo3 cluster may comprise CD206^hi^ CD209^hi^ Mo unrelated cells, we looked at CD206^hi^ CD209^hi^ Mo-like cells strictly defined through their gene co-expression and assessed their differential gene expression regarding other CSF monocyte clusters (**Figure 5E**, mean expression of most differentially expressed genes). This confirmed the proinflammatory polarization of CD206^hi^ CD209^hi^ Mo-like cells, illustrated by higher expression of related markers such as *IL18* and *CCR2*, with CCR2 cells being highly predominant in the CNS (**Figure 5F**) as previously described in EAE model (7). Altogether, these data indicated that CD206^hi^ CD209^hi^ Mo-like cells, once reaching the CNS, may have a pathogenic role partly through antigen presentation, fueling local inflammation.

## Discussion

Myeloid cells play a key role in the MS course. Although their infiltration inside the CNS contributes to inflammation, some protective subsets have also been described. Using mass cytometry and scRNA-seq analysis, we highlighted here an enrichment of a peculiar classical monocyte subset (i.e., CD206^hi^ CD209^hi^ Mo cells) in some RRMS patients’ blood at diagnosis, defining a patients’ subgroup displaying a poorer prognosis 2 years following diagnosis. Single-cell RNA-seq analysis pointed out the potential pathological role of this myeloid subset through their CSF enrichment, underlying specific trafficking, and their propensity to process and present antigen.

CD206^hi^ CD209^hi^ Mo cells can be distinguished from other classical monocytes through their high expression of CD206 and CD209, two markers classically expressed by tissue-infiltrating monocyte-derived cells. CD206 and CD209 markers strict co-expression was not described previously in circulating cells; however, monocyte-derived macrophages treated with IL-3 demonstrate an upregulation of Dectin-1, CD206, and in 10% of them, CD209 (52). This phenotype was associated with an increased phagocytosis capacity. Phagocytosis is an important process for antigen processing and presentation to CD4 T cells; in lymph nodes, monocytic cells expressing CD14, CD206, and CD209 have been described as specifically located in the T-cell area and display antigen presentation capacities (53). This is in line with the CD206^hi^ CD209^hi^ Mo-like cell phenotype described in our scRNA-seq dataset, with a high propensity to process and present antigens. Focusing on CD209-expressing CD14 monocytes, it was demonstrated that they are enriched within an inflamed microenvironment where they participate in MS disease activity by supporting CD4 T-cell activation (9) and in rheumatoid arthritis and psoriatic arthritis patients through secretion of pro-inflammatory cytokines (54).

On the other hand, CD206-expressing monocytic cells are classically associated with an anti-inflammatory phenotype, as described for *in vitro* differentiated regulatory macrophages (55) or infiltrating CD14^+^ CD206^+^ tumor monocytes, which specifically express Arginase-1, IL-10, and TGFβ (56). Importantly, it was demonstrated that macrophages found at the center of MS brain demyelinating lesions can express both CD206 and the pro-inflammatory marker iNOS. In their settings, iNOS/CD206 co-expression may represent cells transiting from a pro-inflammatory to a non-inflammatory phenotype (48). Recruitment of CD206/iNOS co-expressing macrophages was also described in the lungs of patients with chronic obstructive pulmonary disease, and these cells are associated with disease severity (57). In the same way, blood circulating CD206-expressing CD14 monocytes are observed in patients with more severe idiopathic membranous nephropathy (58). These apparently conflicting results about the polarization of CD206 monocytes highlight their highly plastic phenotype, which may be controlled by environmental cues and interacting cells. In both murine models and *in vitro* experiments, it was observed that polarization of endothelial cells from the BBB may regulate iNOS and Arginase-1 expression in interacting macrophages. Specifically, inflamed barriers were found to trigger the expression of iNOS (59).

Our data suggest potential interactions between CD206^hi^ CD209^hi^ Mo and BBB endothelial cells, illustrated by the presence of CD206^hi^ CD209^hi^ Mo-like cells in the CSF, along with elevated expression of both VCAM-1 and CCR5 expression. Even if VCAM-1 expression is poorly described on monocytes, its upregulation by blood classical monocytes in co-culture with endothelial cells has been reported (60), supporting CD206^hi^ CD209^hi^ Mo interaction with the BBB. Further, CCR5 is found to be expressed by 70% of CD14^+^ monocytic cells infiltrating the CSF regardless of CNS pathology, while only 20% of blood circulating monocytes are expressing it (61). The CCR5/CCL5 axis has also been demonstrated critical in the recruitment of pathological monocytes within the CNS of EAE mice model (3). Importantly, although CNS microenvironment imprinting was observed about CSF CD206^hi^ CD209^hi^ Mo-like cells (*CX3CR1*, *CLEC10A*), these were found transcriptionally closer to monocytes than to BAMs, while being highly different from microglial subsets, indicating that CD206^hi^ CD209^hi^ Mo-like cells are from peripheral origin.

Therefore, considering (i) CD206^hi^ CD209^hi^ Mo cells blood enrichment at diagnosis in patients with a worse outcome, (ii) CD206^hi^ CD209^hi^ Mo-like cells’ peripheral origin and presence in CSF, (iii) CD206^hi^ CD209^hi^ Mo and CD206^hi^ CD209^hi^ Mo-like cells expression of proteins and transcripts involved in antigen presentation and cell co-stimulation, and (iv) CD206^hi^ CD209^hi^ Mo expression of pro-inflammatory and trafficking markers at the protein (CD45RA (47), VCAM-1, CCR2, CCR5) and transcripts level (*CCR2, IL18*)—prompted us to suggest that CD206^hi^ CD209^hi^ Mo cells represent an activated and pathogenic subset of classical monocyte population that had experienced tissue trafficking. Whether these cells observed by mass cytometry indeed represent a blood recirculating fraction defining CD206^hi^ CD209^hi^ Mo enriched patients nevertheless still has to be confirmed.

Interestingly, we found that patients carrying the MS-associated susceptibility alleles HLA-DRB1*15:01 and HLA-DQB1*06:02 were more frequent among CD206^hi^ and CD209^hi^ Mo patients. The frequency of the HLA-DRB1*15:01 haplotype in MS patients compared to the general population has already been shown to be overrepresented among the MS population (50,48% vs. 24.14% in controls) (62). Although we found several HLA-DRB1*15:01 positive individuals among the MS patients wo CD206^hi^ CD209^hi^ Mo in line with literature regarding the MS population (45%), we observed that 75% of MS patients w CD206^hi^ CD209^hi^ Mo carry this susceptibility gene. We may therefore wonder whether this specific haplotype favors CD206^hi^ CD209^hi^ Mo proliferation and/or survival, partly explaining their specific enrichment in MS. Although B cells and myeloid cells share an overlapping immunopeptidome, HLA-DRB1*15:01-derived self-peptides presentation involved in autoreactive T-cell amplification seems restricted to B cells (63). Nevertheless, CD206^hi^ CD209^hi^ Mo/T-cell interaction through other myelin-derived peptide presentations may participate in disease evolution/relapses as highlighted in mice model (64) and in line with the higher propensity of CCR2 circulating myeloid cells to contact tissue lesions infiltrating T cells in EAE mice (16). Over HLA-DRB1*15:01 expression in monocytes compared to other haplotypes is linked to the specific hypomethylation of the HLA-DRB1*15:01 exon 2 DNA sequence, linking epigenetic HLA-DRB1*15:01 expression and MS risk (65). Whether this mechanism is involved in monocyte/T-cell enhanced interaction supporting T-cell pathological activity or whether demethylation of this gene impacts related genes expression (66) contributing to monocyte pathogenicity hence has to be studied.

Altogether, the combination of (i) leading-edge techniques such as mass cytometry and scRNA-seq performed on (ii) a highly detailed cohort of patients at diagnosis who (iii) benefit from a cautious annual follow-up, allowed us to proceed to an unprecedented description of a circulating myeloid population associated to the patients ‘outcome. However, although the number of patients was enough to draw robust conclusions, this study was highly descriptive, and the lack of a validation cohort to confirm our findings is a limitation of this work. Assessing more patients and controls over a longer period should help in the estimation of CD206^hi^ CD209^hi^ Mo’s contribution to patients’ outcomes, independently of treatments and other confounding factors. A study of patients benefiting from anti-VLA4 treatment would be of particular interest in this context. Further, many conclusions are gene-based as the supportive role of CD206^hi^ CD209^hi^ Mo cells toward T cells has thus to be specifically tested on sorted cells as their potential contribution to pathogenesis through cytokines production. We believe that getting more insight into this cell subset, for example, by blocking their polarization, their trafficking to the CNS, or their antigen presentation process, should be considered as therapeutic strategies.

## Data Availability

The datasets analyzed for this study can be found in the European Genome-Phenome Archive: EGAD50000000445.
