# Supplementary material for: Blood immunophenotyping of multiple sclerosis patients at diagnosis identifies a classical monocyte subset associated to disease evolution
Source: Front Immunol. 2025 Jan 8;15:1494842. doi: 10.3389/fimmu.2024.1494842 (PMC11751469; doi:10.3389/fimmu.2024.1494842)
Supplement: Supplementary file 1 [file DataSheet1.docx]

# Supplemental Table and figure legends

| **Target** | **Clone** | **Company** | **Isotope** | **Localization** |
| --- | --- | --- | --- | --- |
| **CD326 (EpCAM)** | 9C4 | Biolegend | 141Pr | Cell-surface |
| **CD19** | HIB19 | Biolegend | 142Nd | Cell-surface |
| **HLA-DR** | 10.1 | Biolegend | 143Nd | Cell-surface |
| **CD16** | B73.1 | Biolegend | 145Nd | Cell-surface |
| **CD64** | L243 | Biolegend | 146Nd | Cell-surface |
| **CD11c** | 3.9 | Biolegend | 147Sm | Cell-surface |
| **CD33** | WM53 | Biolegend | 148Nd | Cell-surface |
| **CD209** | 9E9A8 | Biolegend | 149Sm | Intracellular |
| **CD14** | M5E2 | Biolegend | 150Nd | Cell-surface |
| **CD123 (IL-3R)** | 6H6 | Biolegend | 151Eu | Cell-surface |
| **CD21** | Bu32 | Biolegend | 152Sm | Cell-surface |
| **CD192 (CCR2)** | K036C2 | Biolegend | 153Eu | Cell-surface |
| **CD163** | GHI/61 | Biolegend | 154Sm | Cell-surface |
| **CD36** | 5-271 | Biolegend | 155Gd | Cell-surface |
| **CD86** | IT2.2 | Biolegend | 156Gd | Cell-surface |
| **CD169** | 7-239 | Biolegend | 158Gd | Cell-surface |
| **CD274 (PD-L1)** | 29E.2A3 | Biolegend | 159Tb | Cell-surface |
| **CD106** | EPR5047 | abcam | 161Dy | Intracellular |
| **CD3** | UCHT1 | Biolegend | 162Dy | Cell-surface |
| **CD49a** | TS2/7 | Biolegend | 163Dy | Cell-surface |
| **CD80** | 2D10 | Biolegend | 165Ho | Cell-surface |
| **CD1a** | HI149 | Biolegend | 167Er | Cell-surface |
| **CX3CR1** | 2A9-1 | Biolegend | 168Er | Cell-surface |
| **CD32** | FUN-2 | Biolegend | 169Tm | Cell-surface |
| **CD54** | HA58 | Biolegend | 170Er | Cell-surface |
| **CD195 (CCR5)** | J418F1 | Biolegend | 171Yb | Cell-surface |
| **CD206 (MMR)** | 15-2 | Biolegend | 172Yb | Cell-surface |
| **S100A9** | A15105J | Biolegend | 173Yb | Intracellular |
| **CD45RA** | HI100 | Biolegend | 174Yb | Cell-surface |
| **CD172a (SIRPa)** | 15-414 | Biolegend | 175Lu | Cell-surface |
| **CD68** | Y1/82A | Biolegend | 176Yb | Intracellular |
| **CD11b (Mac-1)** | ICRF44 | Fluidigm | 209Bi | Cell-surface |
| **CD45** | HI30 | Fluidigm | 89Y | Cell-surface |

Supplemental table 1**:** cytof panel used detailing the antibody clones used, the provider and the metal association

# Supplemental legends

Supplemental Figure 1:

**A:** viSNE illustrating lineage marker expression (from left to right: CD45, CD19, HLA-DR, CD36, CD3) supporting immune cell population discrimination. **B:** Dotplots displaying frequencies of myeloid **(left)**, T cells **(middle)** and B cells **(right)** among peripheral blood mononuclear cells from HC or MS patients, blue symbols illustrate patients corresponding to MS w CD206^hi^ CD209^hi^ Mo.

Supplemental Figure 2:

**A**: Pie chart illustrating HC, MS patients wo CD206^hi^ CD209^hi^ Mo and MS patients w CD206^hi^ CD209^hi^ Mo myelocytic profile as defined through the identified myeloid clusters. Subsets median frequencies are represented for each donor groups **B:** Bubble plot figuring markers expression level (blue scale) and the proportion of cells expressing the related marker within the cluster (dot size).

Supplemental Figure 3:

**A**: Dotplot figuring cMo among myeloid blood circulating cells in HC donors and in MS patients wo CD206^hi^ CD209^hi^ Mo and MS patients w CD206^hi^ CD209^hi^ Mo. **B**: Boxplots illustrating sIL2RA, IL-15, CXCL10, CCL2, CCL20, CXCL12 plasmatic concentration in HC, MS patients with CD206^hi^ CD209^hi^ Mo and MS patients without CD206^hi^ CD209^hi^ Mo. Mann-Whitney test was done to assess significancy. **C:** Graphical representation of CD206^hi^ CD209^hi^ Mo frequencies among myeloid circulating cells associated to donors age at sampling in HC (green diamonds, correlation coefficient: r=-0.05, p=0,82) and both MS groups: enriched (MS w CD206^hi^ CD209^hi^ Mo, red squares, correlation coefficient: r=-0.09, p=0,76) and unenriched (MS wo CD206^hi^ CD209^hi^ Mo, blue circles, correlation coefficient: r=-0.07, p=0,64).

Supplemental Figure 4:

Dotplot illustrating plasmatic neurofilament light chain patients’ content according to their CD206^hi^ CD209^hi^ Mo cells frequency. Concentrations are in pg/mL and Mann-Whitney test was done to assess significancy.

Supplemental Figure 5:

UMAPs figuring events coexpressing *DC-SIGN* (CD209) and *MRC1* (CD206) **(red dots)** in CSF and peripheral blood of each patient.
