# Supplementary figures and images for: Blood immunophenotyping of multiple sclerosis patients at diagnosis identifies a classical monocyte subset associated to disease evolution
Source: Front Immunol. 2025 Jan 8;15:1494842. doi: 10.3389/fimmu.2024.1494842 (PMC11751469; doi:10.3389/fimmu.2024.1494842)

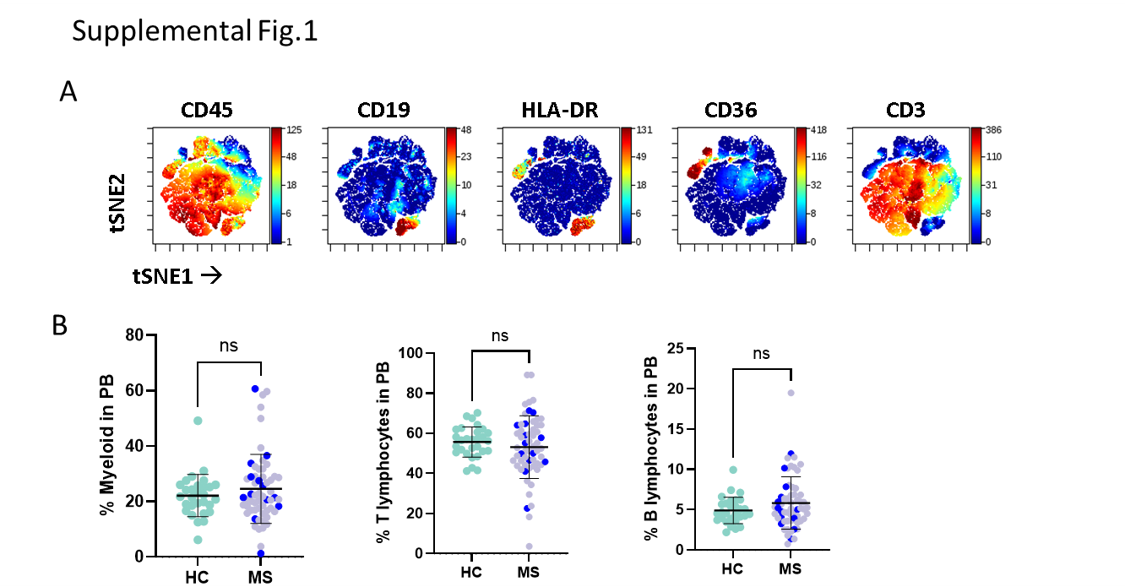

Supplement: Supplementary file 2 [file Image1.tif]

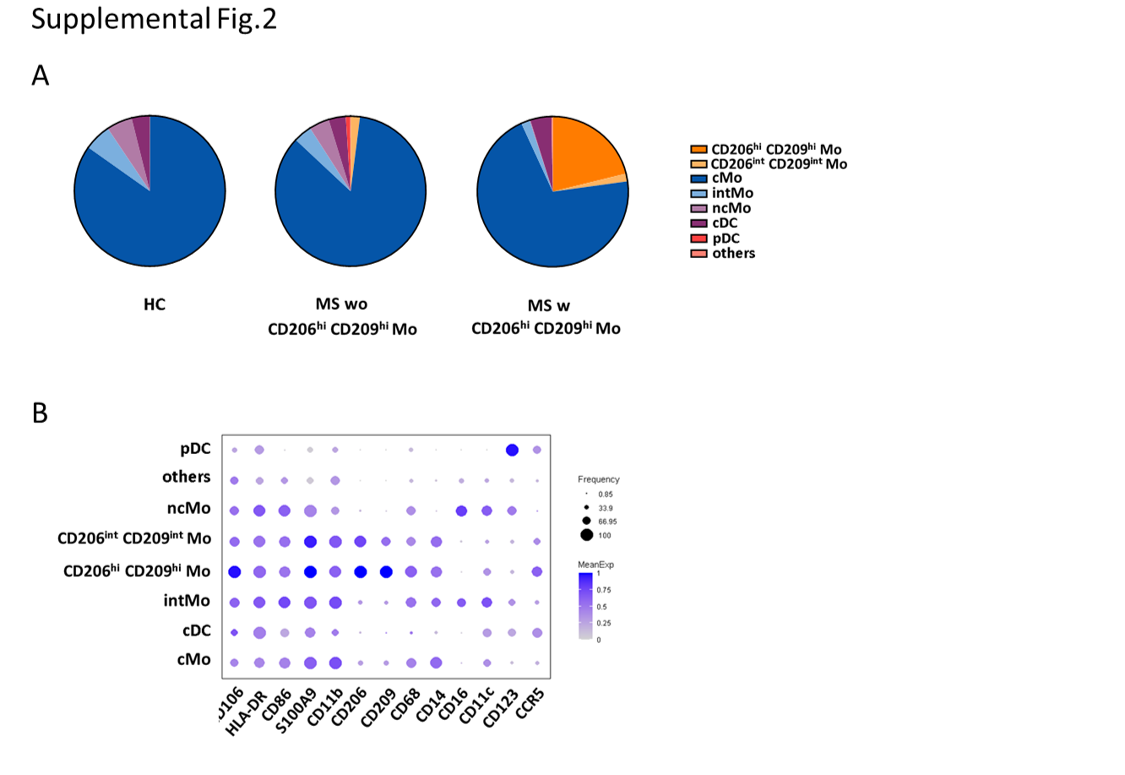

Supplement: Supplementary file 3 [file Image2.tif]

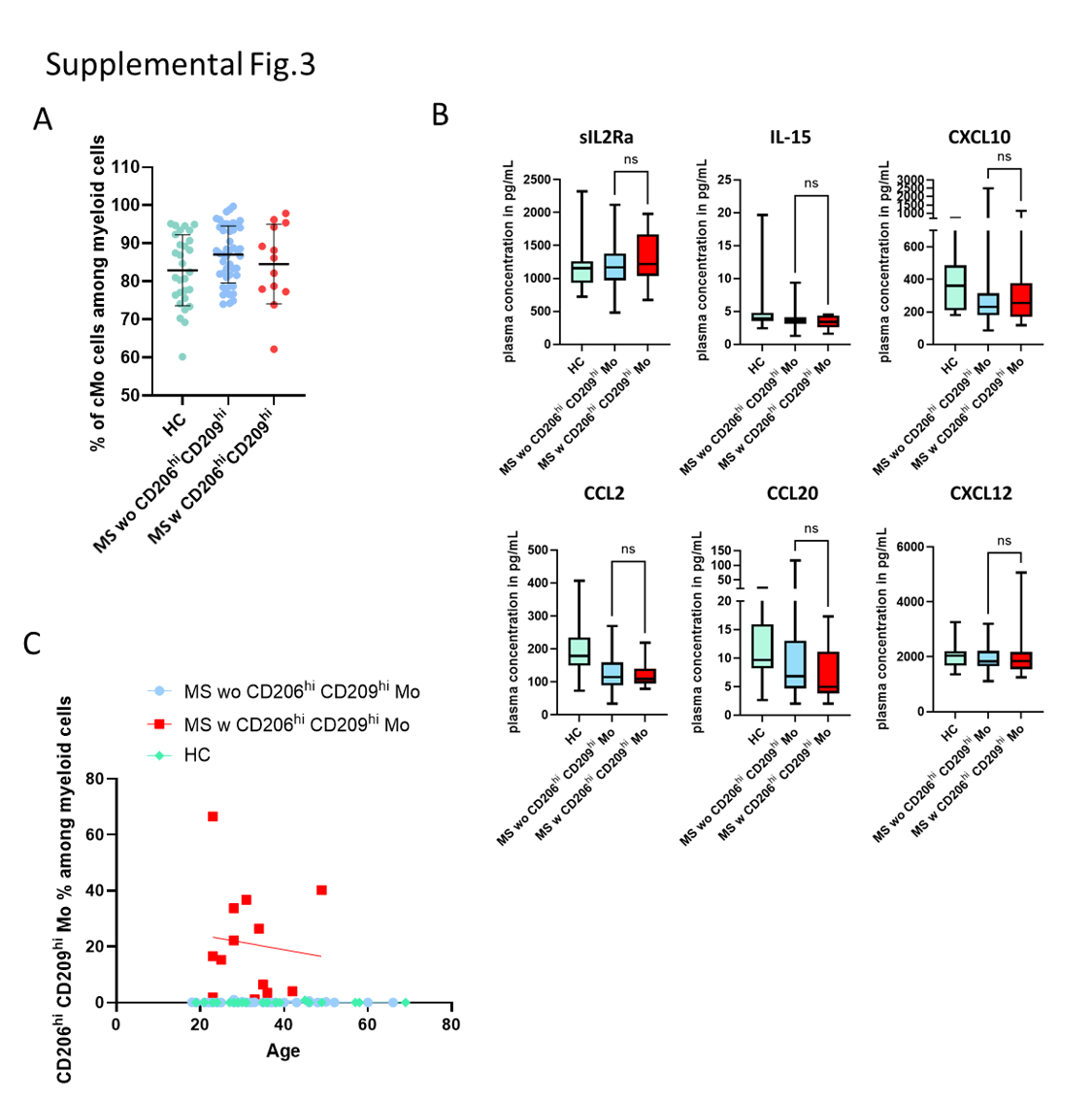

Supplement: Supplementary file 4 [file Image3.tif]

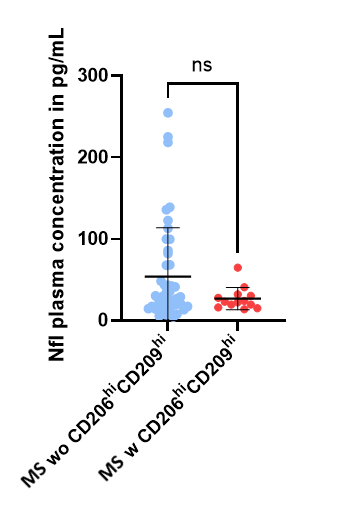

Supplement: Supplementary file 5 [file Image4.tif]

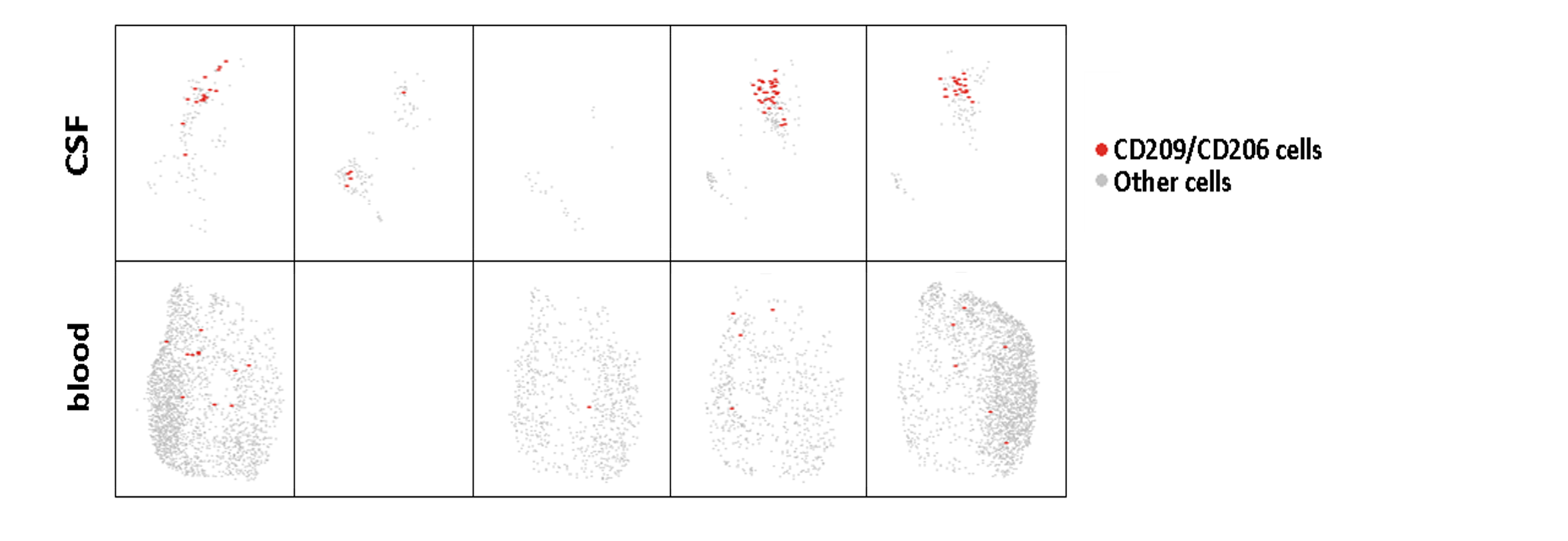

Supplement: Supplementary file 6 [file Image5.tif]
